# Using machine learning to identify risk factors for pancreatic cancer: a retrospective cohort study of real-world data

**DOI:** 10.3389/fphar.2024.1510220

**Published:** 2024-11-21

**Authors:** Na Su, Rui Tang, Yice Zhang, Jiaqi Ni, Yimei Huang, Chunqi Liu, Yuzhou Xiao, Baoting Zhu, Yinglan Zhao

**Affiliations:** ^1^ West China School of Pharmacy, Sichuan University, Chengdu, China; ^2^ Department of Pharmacy, West China Hospital, Sichuan University, Chengdu, China; ^3^ Department of Biotherapy, Cancer Center and State Key Laboratory of Biotherapy, West China Hospital, Sichuan University, Chengdu, China; ^4^ Institute of Medical Information, Chinese Academy of Medical Sciences/Peking Union Medical College, Beijing, China; ^5^ University of Florida Health Shands Hospital, Gainesville, FL, United States; ^6^ National Chengdu Center for Safety Evaluation of Drugs, State Key Laboratory of Biotherapy, West China Hospital, Sichuan University, Chengdu, China

**Keywords:** pancreatic cancer, machine learning, multivariable logistic regression, risk factors, KRAS gene mutation

## Abstract

**Objectives:**

This study aimed to identify the risk factors for pancreatic cancer through machine learning.

**Methods:**

We investigated the relationships between different risk factors and pancreatic cancer using a real-world retrospective cohort study conducted at West China Hospital of Sichuan University. Multivariable logistic regression, with pancreatic cancer as the outcome, was used to identify covariates associated with pancreatic cancer. The machine learning model extreme gradient boosting (XGBoost) was adopted as the final model for its high performance. Shapley additive explanations (SHAPs) were utilized to visualize the relationships between these potential risk factors and pancreatic cancer.

**Results:**

The cohort included 1,982 patients. The median ages for pancreatic cancer and nonpancreatic cancer groups were 58.1 years (IQR: 51.3–64.4) and 57.5 years (IQR: 49.5–64.9), respectively. Multivariable logistic regression indicated that kirsten rats arcomaviral oncogene homolog (KRAS) gene mutation, hyperlipidaemia, pancreatitis, and pancreatic cysts are significantly correlated with an increased risk of pancreatic cancer. The five most highly ranked features in the XGBoost model were KRAS gene mutation status, age, alcohol consumption status, pancreatitis status, and hyperlipidaemia status.

**Conclusion:**

Machine learning algorithms confirmed that KRAS gene mutation, hyperlipidaemia, and pancreatitis are potential risk factors for pancreatic cancer. Additionally, the coexistence of KRAS gene mutation and pancreatitis, as well as KRAS gene mutation and pancreatic cysts, is associated with an increased risk of pancreatic cancer. Our findings offered valuable implications for public health strategies targeting the prevention and early detection of pancreatic cancer.

## 1 Introduction

Pancreatic cancer (PC) is a leading cause of cancer-related death globally, with a 5-year survival rate of approximately 13% (Siegel et al., 2024; Pourshams et al., 2019). PC has an increasing mortality rate and often results in metastasis due to its subtle early symptoms, so most patients are diagnosed at an advanced stage, which limits treatment options (Park et al., 2021). Although computerized tomography (CT) and magnetic resonance imaging (MRI) are effective at diagnosing pancreatic cancer, the cost of these two techniques is relatively high, which limits their wide use (Yang et al., 2021; Diehl et al., 1998; Lu et al., 1997; Sandrasegaran et al., 2013). Seeking potential risk factors could be conducive to early diagnosis and intervention in the risk population.

Generally, risk factors can be categorized into genetic and hereditary factors, environmental factors, medical conditions, and demographic Factors. Genetic factors play a significant role in developing pancreatic cancer with about 10% of pancreatic cancer cases attributed to inherited genetic mutations (Mario et al., 2018). In addition, previous studies indicated that smoking, obesity, and alcohol consumption are responsible for pancreatic cancer (Mario et al., 2018). There is also compelling evidence that factors like chronic pancreatitis and age are associated with pancreatic cancer (Mario et al., 2018; Yuan et al., 2022). Kirsten rat sarcoma viral oncogene homolog (KRAS) may influence pancreatic cancer development through various metabolic alterations. These alterations include enhanced glucose uptake, differential channeling of glucose intermediates, reprogramming of glutamine metabolism, increased autophagy, and macropinocytosis (Bryant et al., 2014). Current knowledge about risk factors for developing pancreatic cancer is focused mainly on the impact of specific risk factors (Yuan et al., 2022; Maisonneuve and Lowenfels, 2015; Kirkegård et al., 2017). However, PC is caused by multiple factors, and little is known regarding the relative predictive power of different risk factors. Traditional methods for identifying risk factors rely on case-control studies and logistic regression models. However, logistic regression models have limitations in data processing, particularly when dealing with large-scale high-dimensional clinical data (Oosterhoff et al., 2022; Song et al., 2021). To address these limitations, we designed a retrospective cohort study to reveal the relationships between different risk factors and pancreatic cancer based on machine learning.

## 2 Methods

### 2.1 Study setting and data source

A retrospective cohort study was conducted using electronic medical records (EMR) from 1 January 2010, and 31 December 2023, at West China Hospital (WCH), Sichuan University (Chengdu, China). All data were extracted from the hospital EMR. The EMR contains information stored in structured or semistructured formats (e.g., patient demographics, physical examination, laboratory tests, medications, and diagnoses). This study was approved by the Institutional Review Board of WCH in May 2021 (WCH 2021-590), and patient consent was waived.

### 2.2 Study population

We included 1,982 patients who had a kirsten rats arcomaviral oncogene homolog (KRAS) gene testing in WCH between 1 January 2010, and 31 December 2023. Patients who met any of the following criteria were excluded: had a history of other malignancies, had incomplete data or missing important information, or had serious complications or illness. Following inclusion, data loss was minimal due to the low rate of missingness in our data source. Given this low rate, statistical methods for handling missing data were not applied.

### 2.3 Definition of pancreatic cancer

Patients with pancreatic cancer were defined as individuals who met the diagnostic criteria for pancreatic cancer and had a confirmed diagnosis at West China Hospital. The diagnostic criteria included clinical symptoms (such as abdominal pain and jaundice), radiological assessments (CT and MRI), histopathological examination, and blood tests (serum CA19-9>39 U/mL) (Chan et al., 2014; Goonetilleke and Siriwardena, 2007; Ni et al., 2005). These factors were analyzed comprehensively to establish the diagnosis by the doctor.

### 2.4 Independent variable

Previous studies identified some potential risk factors for pancreatic cancer. Based on clinical evidence and biological rationale (Kamisawa et al., 2016; McGuigan et al., 2018), we compiled an extensive list of variables to identify potential risk factors, classifying them into four groups: demographic characteristics (age and sex), living habits (smoking and drinking), non-pancreatic comorbidities (hypertension, diabetes, uarthritis/hyperuricemia, overweight/obesity and hyperlipidemia) and pancreatic-related diseases (pancreatic cysts and pancreatitis). For statistical analysis, chi-square tests were used for normally distributed categorical variables, while Wilcoxon rank-sum tests were used for continuous variables that did not conform to a normal distribution. A p-value of ≤0.05 was considered statistically significant.

### 2.5 Multivariable logistic regression

Multivariable logistic regression analyses were performed to calculate the z-value and p-value of the association between each covariate and pancreatic cancer. This initial screening aimed to identify independent variables significantly associated with the disease (p < 0.05).

Significant variables were then included in the multivariable logistic regression model to further evaluate their effects while accounting for potential confounders. The z-value and p-value were computed to estimate the relationship between each variable and pancreatic cancer within the model. Additionally, we performed pairwise multivariable regression with generalized linear models to assess the synergistic effects of KRAS gene mutation and other factors on pancreatic cancer. All statistical analyses were performed using R (version 4.1.3).

### 2.6 Model construction and shapley additive explanations (SHAP)

All the covariates were included in the machine learning models. Twelve machine-learning methods were tested: extreme gradient boosting (XGBoost), random forest (RF), classification and regression tree (CART), support vector classifier (SVC), adaptive boosting (AdaBoost), gradient boosting, neural network (NN), extremely randomized trees (ExtraTrees), balanced bagging classifier, balanced random forest classifier (BalancedRF), random undersampling boosting (RUSBoost) and easyensemble. All variables were included in these models. A training: testing (80:20) approach was used to compute the final set of model-fit-parameters. RandomizedSearchCV was used to search for the optimal hyperparameters for the 12 models. All machine learning models were constructed using 5-fold cross-validation. The accuracy, precision, F1 score, recall, and area under the receiver operating characteristic (AUROC) curve were used to evaluate model performance. Additionally, SHAP values are a powerful tool for interpreting the predictive outcomes of machine learning models by quantifying the impact of each feature on the model’s predictions. In this study, the SHAP technique was utilized to visualize the relationships between these potential risk factors and pancreatic cancer. We included only positive SHAP values, as our goal was to identify potential risk factors for pancreatic cancer. Positive SHAP values specifically indicate contributions toward an increased risk of pancreatic cancer, aligning with our study’s focus.

## 3 Results

### 3.1 Basic characteristics of the study population

The study involved 1,982 patients in the cohort during the study period. As shown in Table 1, we divided the patients into two groups: a pancreatic cancer group and a nonpancreatic cancer group. The median ages for pancreatic cancer and nonpancreatic cancer groups were 58.1 years (IQR: 51.3–64.4) and 57.5 years (IQR: 49.5–64.9), respectively. The gender imbalance observed in this study was statistically significant (p = 0.002), with a greater proportion of males being found in the nonpancreatic group. Additionally, the pancreatic cancer group exhibited a significantly lower median body mass index (BMI) (22.58, IQR: 20.70–24.07) compared to the nonpancreatic cancer group (23.34, IQR: 20.90–25.39) (*p* = 0.004507). We also found a greater prevalence of smoking in the nonpancreatic cancer group, whereas alcohol consumption did not differ significantly between the groups. Notably, the pancreatic cancer group had a greater frequency of KRAS gene mutation (83.7% vs. 51.3%, *p* < 0.001) and a greater prevalence of pancreatitis (18.6% vs. 0.9%, *p* < 0.001) and pancreatic cysts (6.2% vs. 0.2%, *p* < 0.001) compared to the nonpancreatic cancer group.

**TABLE 1:** Baseline characteristics of the patients.

Variables	Contents	Groups (N = 1982)		χ2/W	P value
		Pancreatic cancer (N = 129)	Nonpancreatic cancer (N = 1853)		
Sex	Male	67	1,178	9.34	0.0022
	Female	62	675		
Age (y)	Mean	58.1 (51.3,64.4)	57.5 (49.5,64.9)		
	(0, 20)	0	5	98,969(W)	0.6427
	(20, 40)	7	154		
	(40, 60)	70	909		
	(60, 80)	51	772		
	(80, 100)	1	13		
Histology	Ductal adenocarcinoma	103	0	-	-
	Not performed	26	0	-	-
BMI (kg/m^2^)	Mean	22.58 (20.70,24.07)	23.34 (20.90,25.39)		
	<24	95	1,182	116,701(W)	0.0045
	28>BMI≥24	32	560		
	≥28	2	111		
Smoking (n)	Yes	38	754	7.86	0.0051
	No	91	1,099		
Drinking (n)	Yes	38	754	2.11	0.3475
	No	91	1,099		
KRAS gene	Mutant	108	951	79.35	<0.001
	Wild	21	902		
Metabolic disease (n)	Yes	54	831	5.31	0.0211
	No	75	1,022		
Overweight/Obesity (n)	Yes	34	671	12.39	0.0004
	No	95	1,182		
Diabetes (n)	Yes	21	230	0.15	0.703
	No	108	1,623		
Hypertension (n)	Yes	28	448	0.67	0.4144
	No	101	1,405		
Hyperlipidemia (n)	Yes	13	63	8.93	0.0028
	No	116	1790		
Uarthritis/Hyperuricemia (n)	Yes	6	45	0.77	0.3799
	No	123	1808		
Pancreatitis (n)	Yes	24	16	153.12	<0.001
	No	105	1837		
Pancreatic cyst (n)	Yes	8	4	58.05	<0.001
	No	121	1849		

### 3.2 Multivariable logistic regression

Table 2 presents the results of multivariable logistic regression analyses assessing the associations between baseline variables and pancreatic cancer status. The receiver operating characteristic (ROC) curve of the multivariable logistic regression model revealed that the AUC of the integrated factors was 0.829 (Supplementary Figure S1). KRAS gene mutation (OR = 9.09, 95% CI: 5.50–15.75, p < 0.001), hyperlipidaemia (OR = 3.37, 95% CI: 1.35–7.86, p = 0.006), pancreatitis (OR = 29.97, 95% CI: 12.93–72.27, p < 0.001), and pancreatic cysts (OR = 17.29, 95% CI: 3.85–97.69, p < 0.001) were significantly correlated with an increased risk of pancreatic cancer. After the screening, KRAS gene mutation, hyperlipidaemia, pancreatitis, and pancreatic cysts were entered into the model as independent variables, and KRAS gene mutation (OR = 8.99, 95% CI: 5.48–15.46, p < 0.001), hyperlipidaemia (OR = 3.46, 95% CI: 1.45–7.65, p = 0.003), pancreatitis (OR = 25.30, 95% CI: 11.46–57.79 p < 0.001), and pancreatic cysts (OR = 21.12, 95% CI: 4.71–119.03, p = 0.0001) were significantly associated with the risk of developing pancreatic cancer (Supplementary Table S1).

**TABLE 2:** Multivariable logistic regression.

Intercept	Estimate	Standard error	z value	P	OR	Confidence interval, CI	
						Lower	Upper
Sex	−0.13	0.28	−0.46	0.6440	0.88	0.50	1.51
Age	0.02	0.01	1.75	0.0795	1.02	1.00	1.04
Smoking	−1.05	0.33	−3.22	0.0013	0.35	0.18	0.66
Drinking	−0.22	0.41	−0.53	0.5973	0.81	0.37	1.83
KRAS gene	−2.21	0.27	−8.26	<0.001	9.09	5.50	15.75
Metabolic disease	−1.09	0.44	−2.45	0.0142	0.34	0.14	0.80
Overweight/Obesity	−0.07	0.40	−0.19	0.8508	0.93	0.43	2.05
Diabetes	0.28	0.37	0.75	0.4511	1.33	0.63	2.73
Hypertension	−0.51	0.28	−1.86	0.0624	0.60	0.34	1.01
Hyperlipidemia	1.22	0.45	2.72	0.0065	3.37	1.35	7.86
Uarthritis/Hyperuricemia	0.84	0.51	1.65	0.0988	2.31	0.78	5.88
Pancreatitis	3.40	0.44	7.78	<0.001	29.97	12.93	72.27
Pancreatic cyst	2.85	0.80	3.55	0.0004	17.29	3.85	97.69

### 3.3 Machine learning algorithm

In this study, we developed 12 machine-learning models to identify risk factors for pancreatic cancer (Supplementary Table S2). Five-fold cross-validation was used to evaluate the performance of the constructed models, and we found that RF, CART, and XGBoost outperformed models of data imbalance processing technology (BalanceBagging, BalanceRF, RUSBoost, and EasyEnsemble) (Supplementary Figure S2). We also assessed their performance using metrics such as the area under the curve (AUC), accuracy, precision, recall, and F1 score. As shown in Supplementary Table S3, XGBoost was the best-performing model (AUC = 0.999, accuracy = 0.994, precision = 1.000, recall = 0.909, F1 score = 0.952). The recall and precision scores of RF and CART models are low, as these models often prioritize achieving higher accuracy by classifying the majority of samples as negative cases. According to the above assessment, XGBoost was chosen as the final machine learning model.

**FIGURE 1:**
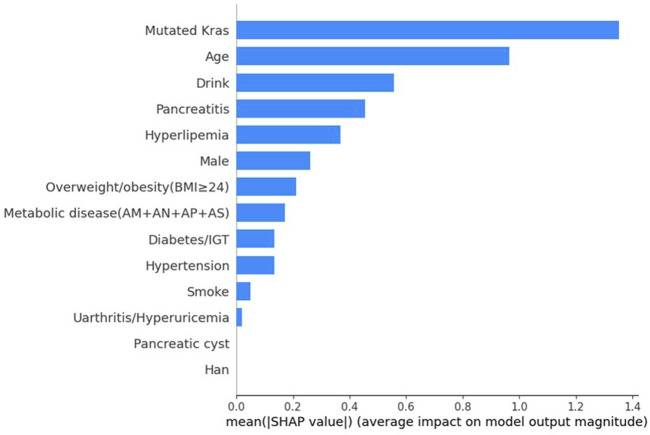
SHAP explanations.

SHAP values indicate the importance of each feature to the prediction of individual instances. We assessed the contributions of different factors in the XGBoost models using SHAP values. Figure 1 displays the importance scores of the different factors. The study identified KRAS gene mutation, age, alcohol consumption status, pancreatitis status, and hyperlipidaemia status as the five most common potential risk factors.

### 3.4 Synergistic effects of KRAS gene mutation and other factors

**FIGURE 2:**
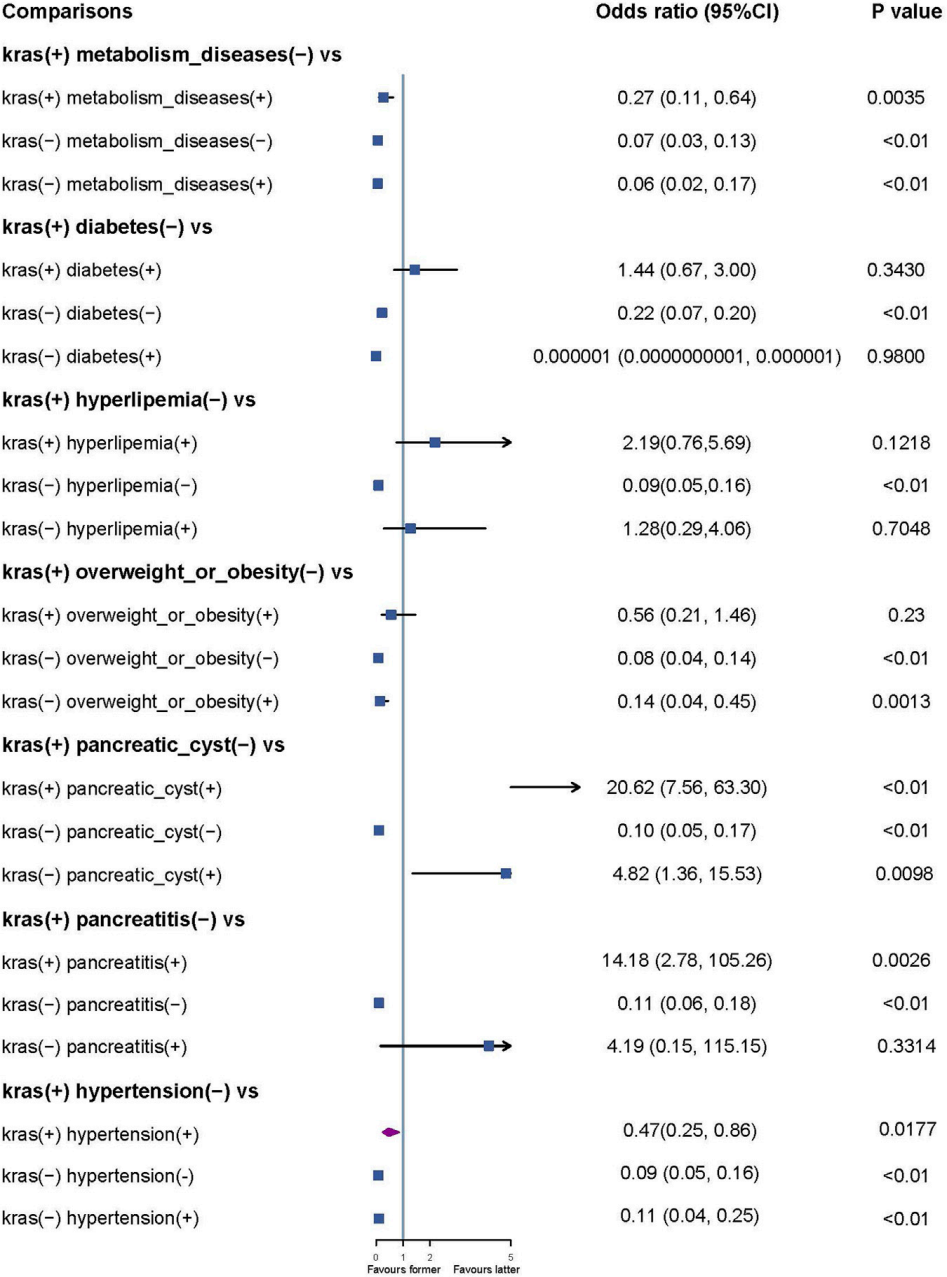
Synergistic effects of KRAS gene mutations and other factors.

Pairwise multivariable regression analyses were conducted to investigate the synergistic effects of KRAS gene mutation and other factors. We found a significant association between the coexistence of KRAS gene mutation and pancreatitis (OR = 14.18, 95% CI: 2.78–105.26, P < 0.01), as well as between KRAS gene mutation and pancreatic cysts (OR = 20.62, 95% CI: 7.56–60.30, P = 0.0026), with an increased risk of pancreatic cancer (Figure 2).

## 4 Discussion

The results of this retrospective cohort study showed that KRAS gene mutation, hyperlipidaemia, pancreatitis, and pancreatic cysts are significantly associated with the risk of developing pancreatic cancer. A machine learning model utilizing demographic characteristics, living habits, nonpancreatic diseases, and pancreatic disease had strong predictive performance (XGBoost, AUC = 0.999). The greatest predictors for pancreatic cancer included KRAS gene mutation, age, alcohol consumption status, pancreatitis, and hyperlipidemia. Both logistic regression and machine learning confirmed that KRAS gene mutation, hyperlipidaemia and pancreatitis are potential risk factors for pancreatic cancer. Additionally, the coexistence of KRAS gene mutation and pancreatitis, as well as KRAS gene mutation and pancreatic cysts, is associated with an increased risk of pancreatic cancer.

The study is among the first to apply advanced machine learning algorithms, specifically XGBoost, to real-world clinical data for the identification of pancreatic cancer risk factors. While previous studies have relied on traditional statistical methods, such as logistic regression, the use of machine learning allows for the handling of high-dimensional data and complex interactions between variables, providing more robust risk prediction models. We also identified the synergistic effects between KRAS mutation and other risk factors, offering new insights into the genetic and biological mechanisms of pancreatic cancer development. Additionally, machine learning models trained on real-world data enable promising applications for improving pancreatic cancer risk assessment, early detection, and diagnosis. However, further validation in diverse populations and prospective clinical studies will be crucial before widespread implementation.

Bryant, Kirsten L. et al. have demonstrated that oncogenic KRAS plays a central role in regulating tumor metabolism, orchestrating diverse metabolic changes such as enhanced glucose uptake, selective channeling of glucose intermediates, reprogrammed glutamine metabolism, increased autophagy, and macropinocytosis (Bryant et al., 2014). Several prior studies have shown similar results: KRAS mutation is related to PC and is found in almost all pancreatic ductal adenocarcinomas (PDACs) (Cox et al., 2014; Luo, 2021). Kamisawa, Terumi et al. reported that KRAS mutation and alterations in *CDKN2A* are early events in pancreatic tumorigenesis (Kamisawa et al., 2016). Bannoura SF et al. suggested that oncogenic KRAS signaling is critical for both the initiation and maintenance of pancreatic cancer; therefore, it is an ideal target for therapy (Bannoura et al., 2021). Although KRAS is a critical oncogene and therefore an important therapeutic target, its therapeutic inhibition is challenging. Recently, specific mutant KRAS inhibitors have been discovered (Bannoura et al., 2021).

Smoking is recognized as a risk factor for many types of cancer (Sasco et al., 2004; Scherübl, 2023). A review and meta-analysis concluded that cigarette smoking causes a 75% increase in the risk of pancreatic cancer compared to nonsmokers, and the risk persists for a minimum of 10 years after smoking cessation (Iodice et al., 2008). Similarly, a meta-analysis indicated that pancreatic cancer risk increases sharply with a low number of cigarettes smoked or after a 5 years of smoking and that it rapidly decreases a few years after cessation, although it takes almost 20 years to reach that of nonsmokers (Lugo et al., 2018). However, we did not find the same result, probably because of bias and the limited study population.

A growing body of evidence suggests that longstanding preexisting chronic pancreatitis is a strong risk factor for pancreatic cancer (Kamisawa et al., 2007; Dítĕ et al., 2010; Kudo et al., 2011). Although there is a strong link between chronic pancreatitis and pancreatic cancer, over 20 years, only approximately five percent of patients with chronic pancreatitis will develop pancreatic cancer (Raimondi et al., 2010). Lin et al. confirmed that hyperlipidaemia can promote tumor growth and subcutaneous tumor formation in mice, and Roy et al. described a two-way relationship between pancreatic cancer and diabetes, which might indicate that there is a complicated relationship between metabolic disease and pancreatic cancer (Qin et al., 2023; Roy et al., 2021).

Identifying risk factors for pancreatic cancer offers significant benefits in clinical and public health contexts. Early detection and targeted screening of high-risk populations can improve the proportion of early-stage diagnoses, which is associated with increased survival rates (Grigorescu et al., 2024). Additionally, understanding modifiable risk factors facilitates the development of targeted public health initiatives, such as lifestyle modification programs and genetic counseling, aimed at mitigating risk in susceptible populations.

A potential weakness of this study is the retrospective nature of this cohort. Since retrospective studies rely on existing records that were not originally collected for research purposes, key information is often missing or incomplete (Talari and Goyal, 2020). There may be variations in diagnostic criteria, treatment protocols, or data entry practices that are difficult to account for retrospectively. Medical records may lack detailed information on confounding variables or precise measurements necessary for robust analysis. These limitations can introduce potential biases and restrict the validity and generalizability of study conclusions. Several statistical methods were used to control for confounding factors; however, some unmeasured residual confounding factors were likely present. Furthermore, due to data inaccuracies and incomplete data, misclassification bias was not uncommon in retrospective database studies. The strength of inference on causality was thus weakened given the retrospective nature of the study. Future studies could address these limitations by implementing strategies such as improving data collection processes, refining study designs, and employing advanced analytical approaches. These enhancements may help to mitigate data gaps, reduce bias, and strengthen the reliability of study findings (Popovic and Huecker, 2024; Jager et al., 2020).

## 5 Conclusion

We confirmed that KRAS gene mutation, hyperlipidaemia, pancreatitis, and pancreatic cysts are significantly correlated with an increased risk of pancreatic cancer. KRAS gene mutation, age, alcohol consumption status, pancreatitis status, and hyperlipidaemia status are the strongest predictors of pancreatic cancer. Both logistic regression and machine learning algorithms confirmed that KRAS gene mutation, hyperlipidaemia and pancreatitis are potential risk factors for pancreatic cancer. Additionally, the coexistence of KRAS gene mutation and pancreatitis, as well as KRAS gene mutation and pancreatic cysts, is associated with an increased risk of pancreatic cancer.

## Data Availability

The original contributions presented in the study are included in the article/Supplementary Material, further inquiries can be directed to the corresponding author.
