# Supplementary material for: Using machine learning to identify risk factors for pancreatic cancer: a retrospective cohort study of real-world data
Source: Front Pharmacol. 2024 Nov 21;15:1510220. doi: 10.3389/fphar.2024.1510220 (PMC11617206; doi:10.3389/fphar.2024.1510220)

***Supplementary Material***

**Using machine learning to identify risk factors for pancreatic cancer: A retrospective cohort study of real-world data**

**Na Su ^1, 2, 3^, Rui Tang ^4^, Yice Zhang ^1^, Jiaqi Ni ^1^, Yimei Huang ^5^, Chunqi Liu ^3, 6^, Yuzhou Xiao ^3, 6^, Baoting Zhu ^1^, Yinglan Zhao ^3, 6*^**

1 West China School of Pharmacy, Sichuan University, Chengdu, China

2 Department of Pharmacy, West China Hospital, Sichuan University, Chengdu, China

3 Department of Biotherapy, Cancer Center and State Key Laboratory of Biotherapy, West China Hospital, Sichuan University, Chengdu, China.

4 Institute of Medical Information, Chinese Academy of Medical Sciences/Peking Union Medical College, Beijing, China

5 University of Florida Health Shands Hospital, Gainesville, Florida, USA.

6 National Chengdu Center for Safety Evaluation of Drugs, State Key Laboratory of Biotherapy, West China Hospital, Sichuan University, Chengdu, China.

***** **Correspondence:**

Yinglan Zhao, Department of Biotherapy, Cancer Center and State Key Laboratory of Biotherapy, National Chengdu Center for Safety Evaluation of Drugs, State Key Laboratory of Biotherapy, West China Hospital, Sichuan University. Sichuan University, 17#, 3rd Section, Ren min South Road, Chengdu 610041, China. Phone: +86-28-85502796; Fax: +86-28-85502796; E-mail: zhaoyinglan@scu.edu.cn

Supplementary Table 1. Multivariable logistic regression after screening variables

| Intercept | Estimate | Standard Error | z value | P | OR | Confidence Interval, CI | |
| --- | --- | --- | --- | --- | --- | --- | --- |
|  |  |  |  |  |  | Lower | Upper |
| Smoking | -0.89 | 0.23 | -3.93 | <0.001 | 0.41 | 0.26 | 0.63 |
| KRAS gene | 2.20 | 0.26 | 8.33 | <0.001 | 8.99 | 5.48 | 15.46 |
| Metabolic disease | -0.97 | 0.22 | -4.36 | <0.001 | 0.38 | 0.24 | 0.58 |
| Hyperlipidemia | 1.24 | 0.42 | 2.95 | 0.0032 | 3.46 | 1.45 | 7.65 |
| Pancreatitis | 3.23 | 0.41 | 7.86 | <0.001 | 25.30 | 11.46 | 57.79 |
| Pancreatic cyst | 3.05 | 0.80 | 3.80 | 0.0001 | 21.12 | 4.71 | 119.03 |

Supplementary Table 2. Basic characteristics of the included population and different data sets

|  | All | Training set | Test set | χ2/W | P-value |
| --- | --- | --- | --- | --- | --- |
| Age | 57.60[49.73,64.90] | 57.50[49.80,64.70] | 58.10[49.60,65.30] | 0.052(W) | 0.447 |
| Male | 1101 | 873 | 228 | 0.882 | 0.348 |
| Smoke | 707 | 560 | 147 | 0.432 | 0.511 |
| Drink |  |  |  | 0.211 | 0.900 |
| Occasionally | 379 | 303 | 76 |  |  |
| Frequently | 276 | 218 | 58 |  |  |
| Han | 1656 | 1323 | 333 | 0.206 | 0.650 |
| Mutated KRAS | 779 | 627 | 152 | 0.245 | 0.620 |
| Diabetes | 251 | 196 | 55 | 0.648 | 0.421 |
| Overweight | 705 | 573 | 132 | 1.293 | 0.256 |
| Hypertension | 427 | 339 | 88 | 0.117 | 0.732 |
| Hyperlipemia | 76 | 59 | 17 | 0.270 | 0.603 |
| Uarthritis | 51 | 38 | 13 | 0.977 | 0.323 |
| Metabolic diseases | 885 | 706 | 179 | 0.042 | 0.838 |
| Pancreatitis | 40 | 32 | 8 | 0.000 | 0.996 |
| Pancreatic cyst | 11 | 8 | 3 | 0.362 | 0.547 |
| Pancreatic cancer | 129 | 107 | 22 | 0.774 | 0.379 |

Supplementary Table 3. Performance evaluation of 12 machine models

| Model | AUC | Accuracy | Precision | Recall | F1 score |
| --- | --- | --- | --- | --- | --- |
| XGBoost | 0.999 | 0.994 | 1.000 | 0.909 | 0.952 |
| RF | 0.991 | 0.956 | 0.818 | 0.409 | 0.545 |
| CART | 0.942 | 0.947 | 0.750 | 0.273 | 0.399 |
| SVC | 0.839 | 0.935 | 0.000 | 0.000 | 0.000 |
| AdaBoost | 0.845 | 0.935 | 0.000 | 0.000 | 0.000 |
| GradientBoosting | 0.967 | 0.962 | 0.909 | 0.455 | 0.606 |
| Neural Network | 0.952 | 0.971 | 1.0 | 0.545 | 0.706 |
| ExtraTrees | 0.833 | 0.935 | 0.000 | 0.000 | 0.000 |
| BalanceBagging | 0.931 | 0.812 | 0.238 | 0.864 | 0.373 |
| BalanceRF | 0.747 | 0.569 | 0.107 | 0.773 | 0.188 |
| RUSBoost | 0.901 | 0.698 | 0.165 | 0.909 | 0.280 |
| EasyEnsemble | 0.972 | 0.736 | 0.196 | 1.000 | 0.328 |

Supplementary Figure 1. The ROC curve of the multivariable logistic regression model (AUC=0.8290114)


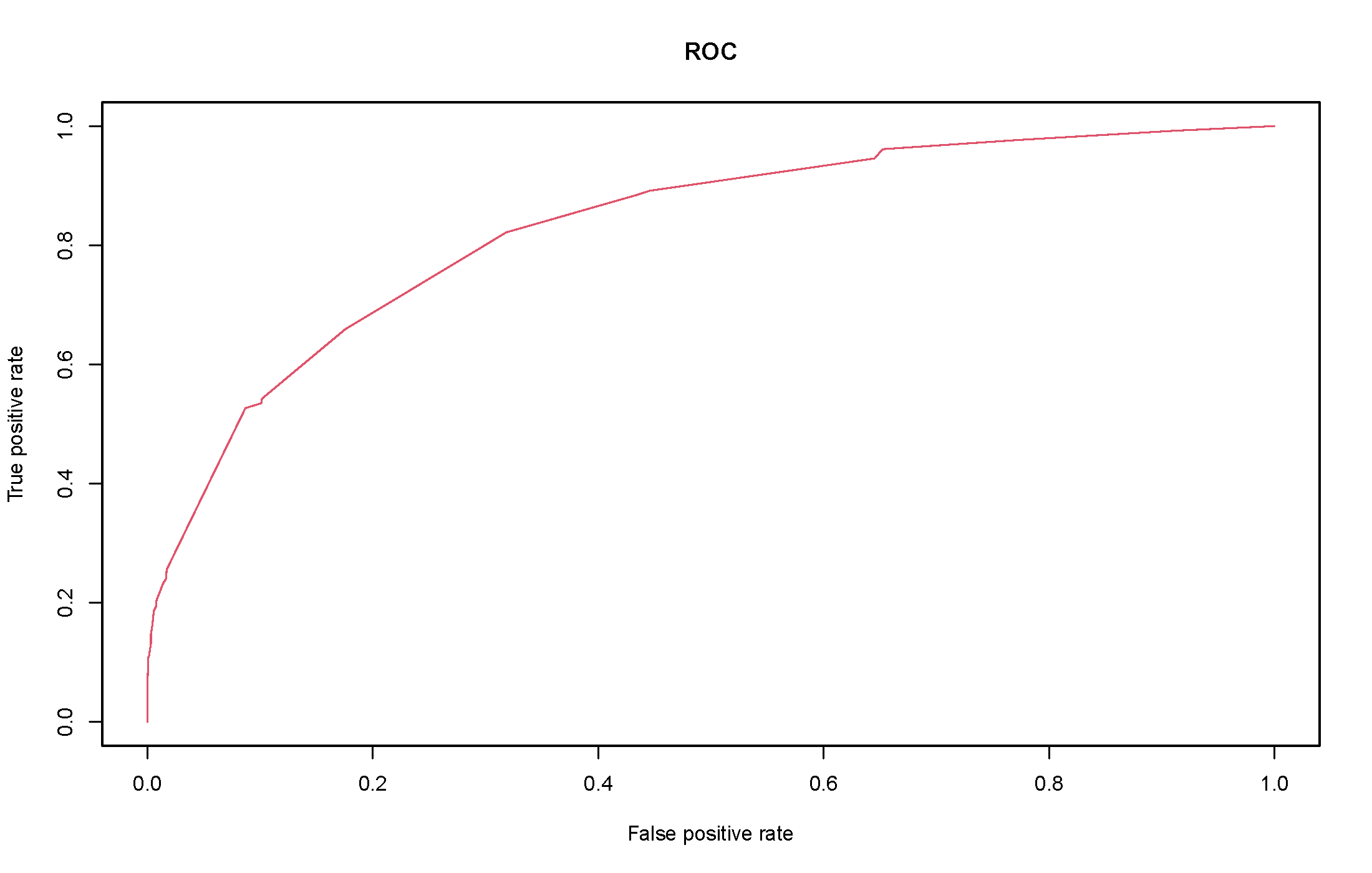


Supplementary Figure 2. Preliminary evaluation of 12 machine learning models in accuracy


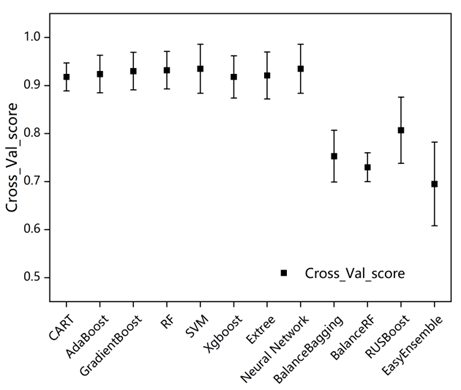

Supplement: Supplementary file 1 [file DataSheet1.docx]
